# Navigating Complexity in Postural Orthostatic Tachycardia Syndrome

**DOI:** 10.3390/biomedicines12081911

**Published:** 2024-08-20

**Authors:** Hui-Qi Qu, Hakon Hakonarson

**Affiliations:** 1 The Center for Applied Genomics, Children’s Hospital of Philadelphia, Philadelphia, PA 19104, USA; quh@chop.edu; 2 Division of Human Genetics, Division of Pulmonary Medicine, Children’s Hospital of Philadelphia, Philadelphia, PA 19104, USA; 3 Department of Pediatrics, The Perelman School of Medicine, University of Pennsylvania, Philadelphia, PA 191104, USA; 4 Faculty of Medicine, University of Iceland, 101 Reykjavik, Iceland

**Keywords:** dysautonomia, genomics, myocardial function, thoracic hypovolemia

## Abstract

Postural Orthostatic Tachycardia Syndrome (POTS) affects up to 1% of the US population, predominantly women, and is characterized by a complex, elusive etiology and heterogeneous phenotypes. This review delves into the intricate physiology and etiology of POTS, decoding the roles of the sinoatrial node, the autonomic nervous system, fluid dynamics, and the interplay between the immune and endocrine systems. It further examines key contributing factors such as dysautonomia, thoracic hypovolemia, autonomic neuropathies, sympathetic denervation, autoimmune responses, and associations with conditions such as small-fiber neuropathy and mast cell activation syndrome. Given the numerous mysteries surrounding POTS, we also cautiously bring attention to sinoatrial node and myocardial function, particularly in how the heart responds to stress despite exhibiting a normal cardiac phenotype at rest. The potential of genomic research in elucidating the underlying mechanisms of POTS is emphasized, suggesting this as a valuable approach that is likely to improve our understanding of the genetic underpinnings of POTS. The review introduces a tentative classification system for the etiological factors in POTS, which seeks to capture the condition’s diverse aspects by categorizing various etiological factors and acknowledging co-occurring conditions. This classification, while aiming to enhance understanding and optimize treatment targets, is presented as a preliminary model needing further study and refinement. This review underscores the ongoing need for research to unravel the complexities of POTS and to develop targeted therapies that can improve patient outcomes.

## 1. Introduction

In the continually evolving landscape of medical intricacies, one condition that commands our attention is Postural Orthostatic Tachycardia Syndrome (POTS). POTS is characterized by a sustained increase in heart rate upon assuming an upright position, devoid of a consequential drop in blood pressure [1,2,3,4]. Specifically, the increase in heart rate (HR) exceeds 30 beats per minute in adults or surpasses 40 beats per minute in individuals aged 12–19 years within 10 min of assuming an upright posture from a lying down or sitting position [5,6]. It is noteworthy that despite this heart rate surge, there is an absence of orthostatic hypotension, defined as a decrease in systolic blood pressure (BP) exceeding 20 mmHg or diastolic BP exceeding 10 mmHg. This phenomenon manifests in a spectrum of symptoms, including dizziness, tachycardia, cognitive dysfunction, fatigue, disrupted sleep, headaches, and gastrointestinal dysmotility. These symptoms collectively exert a profound impact on the lives of those affected.

While the prevalence is estimated between 0.2% and 1.0% in the US population [1,2], the disorder was not officially recognized until the early 1990s [7]. POTS emerges as a distinctive clinical entity within the landscape of dysautonomias. Prior to this recognition, the disparate symptoms often led to misdiagnoses or were attributed to other medical conditions. POTS is more prevalent among women, with a female-to-male ratio of 5:1 [8]. The connection with sex remains poorly understood. One potential link is the increased susceptibility of women to autoimmunity [9]. Furthermore, there is a recognized association between female hormones, especially estrogen, and changes in blood volume and vascular function [10]. While POTS can affect individuals of various ages, a significant proportion of patients are diagnosed between the ages of 15 and 50 [11,12]. This age range encompasses adolescence, young adulthood, and the years leading up to menopause [13]. POTS is rare among either prepubertal girls or postmenopausal women [1]. In the following sections, we will delve deeper into the intricate cardiac physiology, potential etiological factors, and cutting-edge molecular research that seeks to unravel the mysteries surrounding POTS. As we embark on this exploration, we aim to equip medical professionals with a comprehensive understanding of POTS, facilitating improved diagnostic acumen and patient care.

In this review, we chose a narrative approach over a systematic review due to the inherently complex and multifaceted nature of POTS. The etiology and presentation of POTS are highly heterogeneous, with overlapping and interrelated factors that resist the rigid categorization typical of systematic reviews. A narrative review allows for a more comprehensive and nuanced exploration of the condition, integrating diverse perspectives from physiology, genetics, and clinical observations. This format is particularly suited to the current state of POTS research, where many aspects remain speculative or are under active investigation. By synthesizing findings from various domains and proposing tentative classifications and hypotheses, this narrative review aims to provide a holistic understanding of POTS and to highlight areas where further research is needed rather than focusing narrowly on predefined clinical outcomes.

## 2. Heart Rate Regulation: Sinoatrial Node (SAN) and Autonomic Control

The essential components governing the regulation of heart rate, directed by the SAN, and the pivotal role of the autonomic nervous system (ANS) are foundational to cardiovascular control. At the core of this orchestration is the SAN, a cluster of myocytes serving as the natural pacemaker [14]. The SAN’s coordinated generation of electrical impulses dictates the heart’s rate and rhythm. Possessing a heterogeneous pacemaker structure and a dynamically shifting origin of action potential, the SAN conducts a symphony of cardiac activity [15]. The precision of the heartbeat is meticulously fine-tuned by the ANS, where the parasympathetic division decelerates electrical impulses, inducing an inferior shift in the SAN’s leading pacemaker. Conversely, the sympathetic division accelerates impulses, prompting a superior shift [16,17]. This delicate equilibrium ensures the intricate modulation of heart rate in response to physiological demands.

A recent study challenges the conventional paradigm of cardiac pacemaker rate modulation through the introduction of a conceptual framework termed the ‘gear model’. Within this model, the SAN is conceptualized as a brain-like structure characterized by interconnected clusters, each resembling a modular gear. Notably, the ‘gear model’ suggests that specific modules within the SAN respond differentially to autonomic signals, with one module exhibiting sensitivity to β-adrenergic stimulation and suppression by parasympathetic activity, while another module remains less responsive to parasympathetic influence [18]. This modular, gear-like arrangement not only mirrors observed shifts in the leading pacemaker site but also presents a holistic understanding of rate modulation while conserving energy. In the context of POTS, the ‘gear model’ suggests that the balance between these SAN clusters might be disrupted, leading to an inappropriate dominance of the cluster sensitive to β-adrenergic stimulation. This disruption could result in the excessive heart rate increase observed in POTS patients. This heightened response might also make the SAN less adaptable to varying autonomic inputs, which could contribute to the instability in heart rate regulation often experienced by POTS patients. This innovative model suggests the complex interplay between the SAN and the ANS in POTS, potentially uncovering novel pathways for understanding the syndrome’s underlying mechanisms and informing targeted therapeutic interventions.

The transition from a supine to an upright posture triggers substantial hemodynamic shifts, redistributing approximately 500 to 1000 mL of blood volume from the thorax to the lower extremities and splanchnic circulation. Simultaneously, 10% to 25% of plasma undergoes a shift to interstitial fluid [19,20]. These fluid dynamics play a pivotal role in adapting to changes in body position, necessitating a compensatory adjustment in cardiac output. As the body shifts to an upright posture, a decrease in venous return to the heart and stroke volume sets in motion a complex interplay involving both sympathetic and parasympathetic components, orchestrated by baroreceptors and stretch receptors strategically located in cardiovascular structures [21]. Baroreceptors in the carotid sinus and aortic arch, sensing diminished blood pressure, prompt the medulla oblongata to stimulate the sympathetic nervous system while concurrently modulating the parasympathetic branch [22]. Simultaneously, stretch receptors in the heart and lungs sense reduced cardiac filling, influencing both sympathetic and parasympathetic responses [23]. The sympathetic nervous system releases norepinephrine, leading to increased heart rate, enhanced contractility, and peripheral vasoconstriction, aiming to sustain cardiac output and vital organ perfusion. In contrast, parasympathetic activity, mediated by the vagus nerve, acts to counterbalance these effects, promoting relaxation and slowing the heart rate. This intricate interplay between autonomic divisions culminates in a rapid rise of 10–20 bpm in heart rate, peaking within seconds before gradually tapering [19,20].

Moreover, local vascular responses, including changes in blood vessel diameter [24], neurovascular factors with the release of neurotransmitters at the vascular level [25], and neurohumoral factors such as hormonal influences on vascular tone [26], contribute significantly to finely tuned adjustments in blood flow and cardiac function during postural changes. Various intricate responses collectively regulate cardiovascular function during shifts in posture. The endocrine system influences this dynamic regulation through hormonal responses, releasing epinephrine and cortisol to impact heart rate and vascular tone [27]. Simultaneously, the activation of the renin-angiotensin system plays a pivotal role in adjusting blood pressure [28]. Central command mechanisms and peripheral chemoreceptors, responding to oxygen and carbon dioxide levels, further contribute to the nuanced orchestration of cardiovascular adjustments [29]. The skeletal muscle pump aids venous return, and a significantly impaired muscle-pump baroreflex following 60-day bedrest immobilization has been observed [30]. These multifaceted responses collectively ensure the dynamic regulation of cardiovascular parameters, maintaining optimal perfusion and oxygen delivery to tissues.

## 3. POTS Etiology and Its Complexity

**Figure 1:**
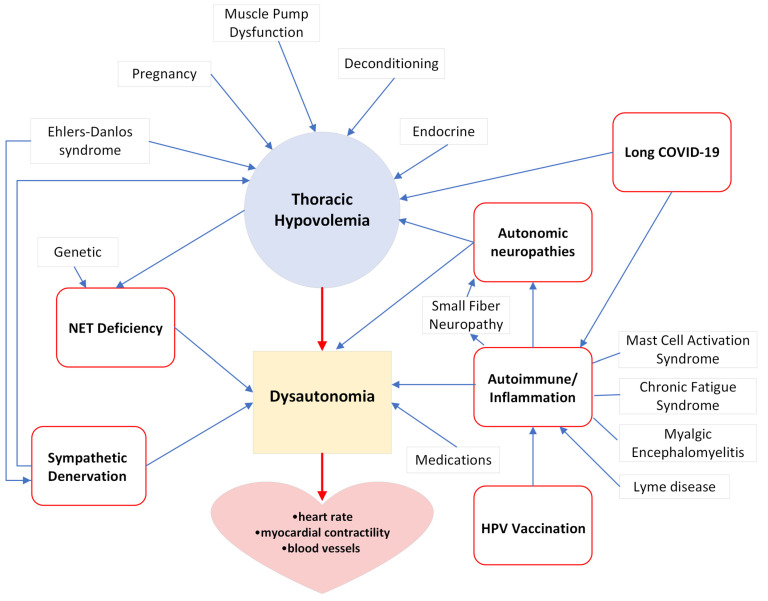
Etiological Factors of POTS. Extended thoracic hypovolemia has the potential to strain the ANS, leading to dysautonomia. Autoimmune responses targeting components of the ANS may trigger dysautonomia. POTS frequently co-occurs with conditions that have inflammatory underpinnings, suggesting inflammatory mechanisms in the pathophysiology of POTS. The diminished venoconstriction resulting from autonomic neuropathies or sympathetic denervation in the lower extremities induces thoracic hypovolemia. The impaired reuptake of norepinephrine due to NET deficiency can result in elevated circulating levels, further contributing to autonomic dysfunction. A decrease in norepinephrine clearance might be attributed to a decline in cardiac output. Molecular mimicry, leading to the formation of cross-reacting autoantibodies, may underlie the risk of POTS associated with HPV vaccination. The mechanisms behind long COVID-19 and POTS may involve hypovolemia, inflammation, and autoimmunity. Additionally, EDS may be linked to POTS due to peripheral neuropathy and thoracic hypovolemia. Endocrine dysfunction, deconditioning, muscle pump dysfunction, mast cell activation disorders, and pregnancy may also contribute to thoracic hypovolemia. Medications may cause dysautonomia. Dysautonomia leads to issues with heart rate regulation, myocardial contractility, and the function of blood vessels. Our research further highlights the significant challenges affecting the heart and circulatory system in POTS patients. Despite the fragmented understanding of POTS risk factors, highlighting a complex interplay of physiological, immunological, and environmental factors, the identification of these diverse factors underscores critical gaps in knowledge, such as the direct involvement of the heart, and offers insights into potential areas for intervention and therapeutic development. Red arrows represent central mechanisms, and blue arrows represent contributing factors.

POTS presents a complex medical challenge, posing difficulties for both patients and healthcare professionals due to its intricate array of symptoms and underlying causes. In the literature, POTS is reported to be more common in Caucasian populations compared to other racial or ethnic groups [31]. However, other races and ethnicities are likely underrepresented, reflecting potential health disparities. On the other hand, this racial disparity may involve a complex interplay of genetic, environmental, and healthcare access factors. Genetically, certain populations may carry variants that influence autonomic nervous system function, blood volume regulation, or cardiovascular response, potentially predisposing them to conditions such as POTS. These genetic factors could be further modulated by environmental conditions, such as diet, stress, and physical activity levels, which can vary significantly across different racial and ethnic groups and impact the expression of genetic traits. In addition to these genetic and environmental factors, healthcare access plays a critical role in the diagnosis and management of POTS. Populations with limited access to specialized healthcare providers, diagnostic tools, and treatment options may be underdiagnosed or misdiagnosed, leading to an underrepresentation of POTS in certain racial or ethnic groups. Moreover, cultural differences in the perception and reporting of symptoms, as well as potential biases within the healthcare system, could contribute to disparities in the recognition and treatment of POTS across different populations [32]. A thorough exploration into the etiology of POTS has unveiled a highly intricate interplay of physiological, immunological, and neurological factors that contribute to the onset and perpetuation of this syndrome (Figure 1).

Dysautonomia: POTS is fundamentally characterized by dysautonomia, an intricate disturbance in the delicate equilibrium between sympathetic and parasympathetic activity [33]. This disruption serves as a pivotal mechanism in symptom manifestation by compromising the precise regulation of heart rate and blood pressure, highlighting the intricate interplay between the sympathetic and parasympathetic branches of the ANS.

Thoracic hypovolemia: While dysautonomia is a core feature, a crucial aspect of understanding POTS is the concept of thoracic hypovolemia, where a reduction in blood volume within the thoracic cavity leads to a diminished venous return [3]. Hypovolemia is associated with factors such as increased splanchnic blood flow, blood pooling in the lower extremities, dehydration, and insufficient fluid intake [3,34]. Notably, the likelihood of POTS increases significantly, or 3.9 times, when a child’s daily water intake is below 800 mL [35]. The administration of intermittent IV infusions of saline has shown a remarkable reduction in symptoms and an improvement in the quality of life for POTS patients [36].

Autonomic neuropathies: Autonomic neuropathies may develop either acutely or chronically. Acute-onset cases are linked to conditions such as paraneoplastic syndromes, Guillain-Barre syndrome, Sjögren disease, infections, or exposure to toxins and chemotherapy. Chronic autonomic neuropathies, on the other hand, are associated with persistent health issues such as diabetes and autoimmune disorders [37]. The dysregulation of the autonomic nervous system by autonomic neuropathies affects blood flow and heart rate control, which may cause POTS.

Sympathetic denervation: Sympathetic denervation, characterized by a decrease in sympathetic nerve activity, is another factor contributing to autonomic dysfunction in POTS [38]. This denervation disrupts the intricate coordination between the nervous and cardiovascular systems, ultimately giving rise to the hallmark symptoms of POTS. Reduced venoconstriction due to sympathetic denervation in the lower extremities causes thoracic hypovolemia. Furthermore, a subset of patients with neuropathic POTS may exhibit mild small-fiber neuropathy, characterized by abnormalities in unmyelinated nerve fibers in the skin. This condition is closely associated with reduced postganglionic sympathetic innervation of the myocardium [39].

Autoimmune: Autoimmune responses directed at components of the ANS or other self-antigens may contribute to the development and progression of POTS, creating an immunological enigma within the etiological framework [40,41]. POTS has a higher prevalence of autoimmune markers and co-morbid autoimmune disorders, e.g., Hashimoto’s thyroiditis and rheumatoid arthritis [42]. The onset of POTS is frequently linked to triggers, such as long COVID-19, the human papillomavirus (HPV) vaccine, infections, or trauma, which can subsequently lead to immunological changes. Furthermore, various autoantibodies—such as ganglionic acetylcholine receptor (gAChR) autoantibodies and G-protein coupled receptor autoantibodies—have been identified in POTS. Targeting some of these autoantibodies with immunomodulatory treatments has demonstrated efficacy in providing relief from symptoms [43,44].

Inflammation: In addition to elevated autoimmune and inflammatory markers in POTS patients [45] and a higher prevalence of autoimmune disorders [42], POTS frequently co-occurs with a range of other conditions that have inflammatory underpinnings, such as: Small Fiber Neuropathy (SFN), characterized by damage to small nerve fibers and inflammatory processes [46]; Mast Cell Activation Syndrome (MCAS), involving excessive inflammatory mediator release [47]; Chronic Fatigue Syndrome (CFS)/Myalgic Encephalomyelitis (ME), associated with immune dysregulation and chronic inflammation [48]; and infections such as Lyme disease [49]. These examples underscore the significant role that immune and inflammatory mechanisms may play in the pathophysiology of POTS.

Norepinephrine Transporter (NET) Deficiency: Mutation of the solute carrier family 6 member 2 gene (*SLC6A2*) causes NET deficiency, which presents with symptoms similar to POTS [50]. Consequently, impaired reuptake of norepinephrine, a key neurotransmitter, can result in elevated circulating levels, further contributing to autonomic dysfunction and the symptoms seen in POTS patients. Acquired abnormal norepinephrine clearance can be due to thoracic hypovolemia and a decrease in cardiac output [51].

Endocrine dysfunction: The endocrine system plays a crucial role in regulating hormones that influence various bodily functions, including blood pressure and heart rate. Abnormalities in the renin-angiotensin-aldosterone system (RAAS) and the adrenal glands have been implicated in POTS [52]. On the other hand, endocrine diseases such as pheochromocytoma can lead to excess production of epinephrine and tachycardia, which are typically excluded in a POTS diagnosis [53].

Human papillomavirus (HPV) Vaccination: controversially, some reports have suggested a potential association between POTS and HPV vaccination [54]. While this link remains debated, a suggested mechanism involves molecular mimicry, where IgG generated by the HPV vaccine is hypothesized to cross-react with G protein-coupled receptors (GPCR) [55,56]. However, the molecular mimicry argument for vaccination as the etiology of POTS is insufficient. The timing and circumstances for this are inappropriate, as patients can have a rapid onset of POTS soon after their HPV vaccine, much too soon for IgG production [54].

Long COVID-19: Long COVID-19 has emerged as a potential sequel of the post-acute sequelae of SARS-CoV-2 infection [57] and encompasses a wide range of new, returning, or ongoing health problems that people can experience for weeks, months, or even years after initially recovering from a COVID-19 infection. Among the multitude of symptoms associated with long-term COVID-19, POTS has emerged as a significant clinical issue, marking a notable increase in prevalence and recognition in the context of the pandemic [58]. The mechanisms behind this association are not fully elucidated but may involve inflammation, microthrombi, and autoimmunity [59,60].

Long COVID-19 triggers an extended inflammatory response, characterized by the continuous production of cytokines and other inflammatory mediators [61]. This prolonged state of inflammation can lead to systemic effects, including damage to the vascular endothelium [62] and dysregulation of the ANS [63], creating fertile ground for the development of conditions such as POTS. Simultaneously, long COVID-19 can cause hypovolemia through various pathways, including decreased intake due to illness or direct viral effects on gastrointestinal systems leading to losses through diarrhea [64]. The resultant decrease in blood volume challenges the body’s ability to maintain adequate blood circulation, especially upon standing, which can trigger or exacerbate POTS symptoms. Moreover, the immune response to COVID-19 can lead to autoimmunity, where the body produces antibodies that mistakenly target the autonomic nervous system [60]. In a study we have been involved in, which systematically profiles the antibody response throughout SARS-CoV-2 infection, we have identified the distinctive features of the SARS-CoV-2-specific IgA response in COVID-19 patients [65,66]. Given IgA’s critical role in mucosal immunity and its potential to contribute to autoimmune responses, there is a theoretical basis for its involvement in the autoimmunity in POTS. Furthermore, IgA-mediated inflammation could exacerbate vascular and autonomic dysfunctions, contributing to the symptomatology of POTS [67].

In addition to the above fundamental factors, various supplementary elements can significantly influence POTS. Examples include, but are not limited to: Ehlers-Danlos syndrome (EDS), which overlaps with POTS, includes symptoms of peripheral neuropathy, hypermobility, anxiety, and pain [68,69,70]. Deconditioning: Insufficient physical activity and deconditioning have been associated with thoracic hypovolemia and POTS [71]. Muscle Pump Dysfunction: Reduced contraction of leg muscles, responsible for pumping blood back to the heart, can exacerbate POTS symptoms [72]. Mast Cell Activation Disorders: Dysregulated mast cell activation, leading to the release of vasoactive substances, is implicated in contributing to POTS symptoms [47]. Pregnancy: Hormonal changes during pregnancy can impact blood volume and vascular tone, potentially causing symptoms similar to POTS [73]. Medications: Medication-induced effects (e.g., sympathomimetics, anticholinergics, β-blocker withdrawal) may cause dysautonomia with symptoms similar to POTS [19].

## 4. Myocardial Function and POTS

**Figure 2:**
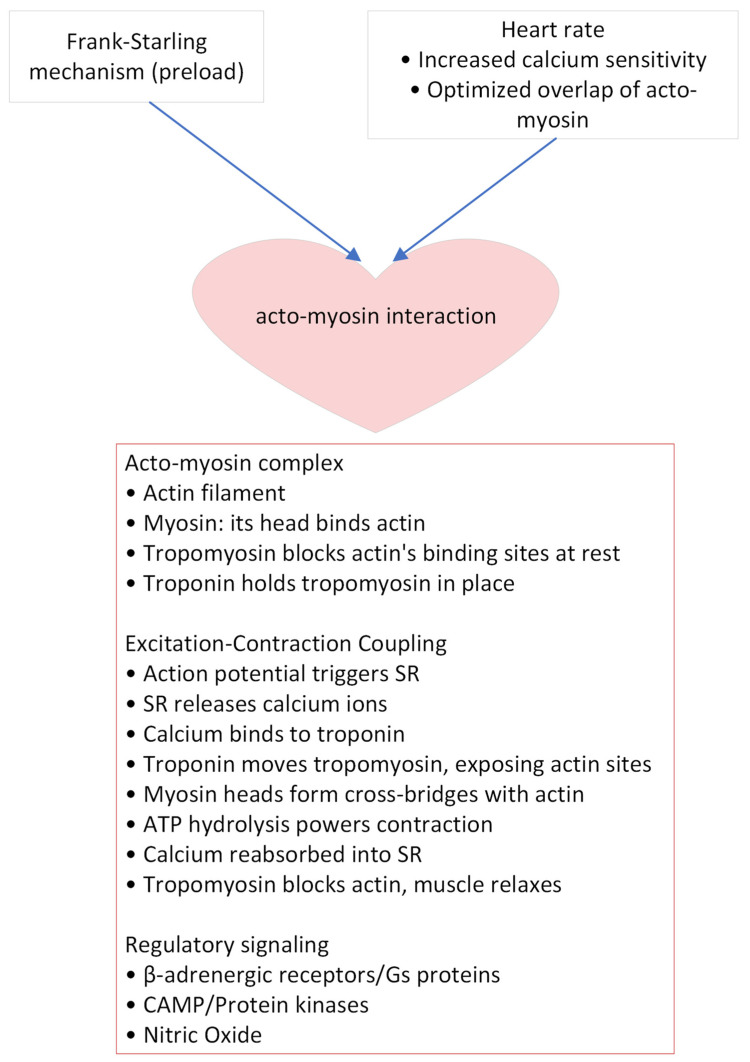
Cardiac contraction and regulatory mechanisms. The purported role of myocardial function in POTS involves a complex interplay of the acto-myosin complex and multiple regulatory mechanisms. Several key players are outlined.

Myocardial function in POTS has received too little research attention to date, particularly regarding subclinical changes without alterations in heart morphology or symptoms. Cardiac contraction is orchestrated through the intricate interaction of the acto-myosin complex and various regulatory mechanisms (Figure 2). At the molecular level, the actin filament and myosin protein interact to form the core of the contraction process. Myosin heads bind to actin, a connection crucial for muscle contraction. This interaction is tightly regulated; tropomyosin blocks the myosin-binding sites on actin filaments in a resting state, while troponin holds tropomyosin in place, ensuring that contraction only occurs upon the appropriate signal. The excitation-contraction coupling mechanism initiates with an action potential that triggers the sarcoplasmic reticulum (SR) to release calcium ions. These ions bind to troponin, causing a conformational change that moves tropomyosin away from the actin binding sites, thereby allowing myosin heads to form cross-bridges with actin. The hydrolysis of ATP then provides the energy necessary for muscle contraction. Subsequently, calcium ions are reabsorbed into the SR, tropomyosin recovers the actin sites, and the muscle relaxes. Regulatory signaling pathways play a pivotal role in modulating cardiac contraction. β-adrenergic receptors, upon activation, interact with Gs proteins, leading to the activation of adenylyl cyclase and the subsequent increase in cyclic AMP (cAMP). This increase in cAMP activates protein kinases, such as Protein Kinase A (PKA), which phosphorylate various proteins involved in calcium handling and contractility, thereby enhancing the heart’s response to stimuli. Additionally, nitric oxide (NO) serves as a key modulator, influencing blood flow and calcium sensitivity in cardiac muscle cells. Collectively, these mechanisms ensure the heart’s ability to effectively respond to varying physiological demands, ensuring the complexity and precision of cardiac muscle regulation [74].

In the context of cardiac efficiency, the interplay of heart rate, myocardial contractility, and cardiac preload and afterload is fundamental. Heart rate directly impacts cardiac output and efficiency, with an optimal rate ensuring adequate ventricular filling and effective stroke volume. Myocardial contractility, the intrinsic ability of cardiac muscle fibers to contract and generate force, is central to the heart’s pumping capacity. Preload, the volume of blood in the ventricles at the end of diastole, influences stroke volume via the Frank-Starling law, where an optimal preload optimizes myocardial fiber stretching for efficient contraction, yet excessive preload can lead to deleterious cardiac dilation and dysfunction. Afterload, the resistance the heart must overcome to eject blood, is largely determined by systemic vascular resistance and arterial pressure. The intricate regulation of these elements is essential for adapting to changing physiological demands, making their understanding pivotal in cardiology.

Blood proteomic studies highlighted the gene set of the actin cytoskeleton. In all muscle types, the actin cytoskeleton is not only essential for contraction but also plays a vital role in cellular signaling, maintenance of cell shape, and intracellular transport. It’s a dynamic and adaptable system critical for the proper functioning of muscles under different physiological conditions [75]. Elevated levels of actin cytoskeleton proteins in the blood suggest compromised functionality in muscles, including the heart and blood vessels. In the meantime, the roles of various muscle types in POTS pathogenesis are increasingly recognized. Skeletal muscles, especially in the lower body, are crucial for venous return through the muscle pump mechanism [30]. Rehabilitation strategies often emphasize the importance of strengthening skeletal muscles [76]. However, the benefits of rehabilitation through exercise for POTS patients extend well beyond the mere strengthening of skeletal muscles. Regular, tailored exercise helps improve heart function and increases cardiovascular efficiency. Over time, this can lead to an increased ability to pump blood effectively, mitigating one of the core challenges faced by POTS patients. The smooth muscles in the vascular system are responsible for maintaining vascular tone. Pharmacological treatments aimed at normalizing vascular tone may be effective for neuropathic POTS, but not for hyperadrenergic POTS [77]. Specifically, cardiomyocytes facilitate the heart’s ability to pump blood throughout the body and are central to the pathophysiology of POTS.

In the study and management of POTS, significant attention has been given to two key aspects: heart rate variability and the cardiac preload. The contractile force exerted by cardiomyocytes (cardiac inotropy) is not typically the primary factor associated with POTS. However, if the inotropy in POTS is altered, it could affect stroke volume. Reduced stroke volume could necessitate a higher heart rate to maintain adequate cardiac output, especially upon standing. Emphasizing the role of cardiac inotropy in POTS challenges the traditional view of POTS and opens new avenues for understanding the pathophysiology of the syndrome.

Cardiac inotropy is influenced by various factors, including neural input (especially sympathetic stimulation [78]), circulating hormones (such as epinephrine [79]), and intrinsic myocardial properties [80]. Gene mutations affecting cardiac inotropy are a significant area of interest in the field of cardiovascular genetics. These mutations can lead to various heart conditions by altering the function of proteins that are crucial for the contraction and relaxation of the heart muscle. Sarcomeres represent the core contractile elements within myocardial fibers, critical for both skeletal and cardiac muscle functionality [81]. These intricate units comprise a network of actin and myosin filaments, pivotal for the contraction mechanism. In the cardiac context, the efficient and synchronized contraction of sarcomeres underpins the heart’s ability to pump blood effectively. This contraction process is intimately associated with excitation-contraction coupling, which translates an electrical signal into mechanical action, involving an orchestration of ion channels, receptors, and signaling cascades [82]. Specifically in cardiomyocytes, this coupling is initiated by an electrical stimulus that prompts calcium ion flux, triggering the actin-myosin interaction within sarcomeres, leading to myocardial contraction.

## 5. Insights into POTS from Omics Studies

Conducting genome-wide association studies (GWAS) for POTS has been particularly challenging due to the disorder’s highly heterogeneous phenotype. This heterogeneity significantly complicates the GWAS process, which traditionally relies on a well-defined, homogeneous phenotype to effectively identify common genetic variants associated with a specific condition. One of the primary hurdles in GWAS for POTS is the precise characterization of its phenotypes. The clinical complexity of POTS complicates the differentiation between its subtypes and the identification of a consistent phenotype that accurately encapsulates the disorder. Additionally, the heterogeneity in POTS can dilute the statistical power of GWAS. The variability within the patient population may obscure the genetic signals that are genuinely associated with the condition, making it more challenging to identify significant genetic associations. This issue is exacerbated in conditions such as POTS, which may not only involve a wide range of symptoms but could also have a complex genetic architecture. Multiple genes, possibly interacting with environmental factors, could contribute to the pathogenesis of POTS, adding layers of complexity to the genetic underpinnings of the disorder.

Recognizing these challenges, our group took an approach by combining GWAS with whole exome sequencing (WES) to explore the molecular mechanisms underpinning POTS pathogenesis. Our GWAS results identified several gene sets associated with various biological processes and disorders, such as substance-related disorders, cell-cell junctions, synaptic membranes, transporter complexes, and early estrogen responses. The WES analysis further highlighted specific genes and molecular mechanisms, including those related to muscular and myocardial dysfunction, as well as mitochondrial activity [83].

Proteomic studies have yielded intriguing results in this area [84,85]. Medic Spahic and colleagues identified growth hormone (GH) and myoglobin (MB) as specific biomarkers for POTS, with POTS patients showing higher plasma levels of GH (especially in women) and lower levels of MB (particularly in men) compared to controls [85]. This suggests that sex-specific immune-neuroendocrine dysregulation and deconditioning may be crucial in the pathophysiology of POTS. Johansson et al. reported 30 proteins in the plasma that were differentially expressed (DEX) in POTS patients compared to healthy controls, highlighting networks involved in thrombogenicity, platelet activity, inflammation, cardiac function, and adrenergic activity [84].

Specifically, among the 30 DEX proteins, six upregulated proteins (MYL1, MYL12B, ILK, PARVB, CAVIN2, and WDR1) are in the gene set of the actin cytoskeleton (GO:0015629, FDR = 0.058). Four of these (MYL1, MYL12B, ILK, and PARVB) are in the gene set of contractile fiber (GO:0043292, FDR = 0.069 [84]). Elevated levels of these actin cytoskeleton proteins could reflect changes in muscle contractility and vascular tone in POTS patients.

Myosin light chain 1/3, skeletal muscle isoform (*MYL1*): This gene encodes a myosin light chain specific to fast-twitch skeletal muscle fibers [86]. It plays a critical role in muscle contraction by modulating the interaction between myosin heads and actin filaments [86]. The dysregulation of *MYL1* may contribute to altered muscle contraction efficiency in POTS.

Myosin regulatory light chain 12B (*MYL12B*): *MYL12B* encodes a regulatory light chain of myosin II [87], primarily found in smooth muscle and non-muscle cells [88]. It thus plays a crucial role in smooth muscle contraction and various cellular movements. In POTS, the dysfunction of *MYL12B* could impair vascular smooth muscle contraction, leading to abnormal blood vessel responses during orthostatic challenges, which might explain some of the cardiovascular instability seen in POTS patients.

Integrin-linked protein kinase (*ILK*): *ILK* is a key component in cell-matrix adhesion and integrin-mediated signal transduction [89]. It connects integrins to the actin cytoskeleton and is involved in the regulation of cell shape, motility, and the cell cycle [90]. *ILK* has been implicated in improving cardiac function [91]. In POTS, altered *ILK* function could impact both cardiac and vascular responses, potentially contributing to the characteristic tachycardia and other cardiovascular symptoms.

Beta-parvin (*PARVB*): Beta-parvin is a component of focal adhesions, structures that link the extracellular matrix to the cytoskeleton [92,93]. It interacts with integrins and actin-binding proteins, playing a role in maintaining vascular integrity and responding to mechanical stress [94]. The adapter protein ParvB inhibits ILK-mediated oncogenic signaling [95]. In POTS, disruptions in *PARVB* function could lead to compromised vascular integrity and abnormal stress responses.

Caveolae-associated protein 2 (*CAVIN2*): This gene is involved in the formation and function of caveolae [96], which are small, flask-shaped invaginations in the plasma membrane. Caveolae are involved in various cellular processes such as signal transduction, lipid regulation, and endocytosis [97,98], with important roles in endothelial cell signaling and vascular function [99]. Altered *CAVIN2* expression in POTS could disrupt endothelial cell signaling and vascular function.

WD repeat-containing protein 1 (*WDR1*): *WDR1* is involved in actin dynamics [100]. It promotes actin filament depolymerization, which is vital for cell shape changes and motility [101], indicating its potential impact on vascular and possibly immune system responses in POTS. Mutations in *WDR1* have been linked to diseases involving the hematological and immunological systems [101]. In POTS, dysregulation of *WDR1* might affect vascular tone and immune responses.

Clinically, abnormalities in echocardiograms are uncommon among patients with POTS, with ejection fractions typically falling within the normal range [1]. On the other hand, our research by GWAS and WES, alongside the reported proteomics analysis by others, has highlighted gene sets pertinent to myocardial function. The prominence of these gene sets in our studies indicates that while POTS patients may exhibit a phenotype of normal cardiac function without evident pathology at rest, there may be underlying genetic predispositions that affect the heart’s response to stress. Furthermore, identifying biomarkers for POTS could pave the way for new targeted therapies centered on altering specific molecular pathways. For instance, modulating integrin-linked pathways may improve vascular responses. Additionally, POTS biomarkers could aid in developing precision medicine strategies for POTS, allowing for treatments that are customized according to each patient’s unique genetic makeup. This can be particularly beneficial given the heterogeneity of the condition.

## 6. Clinical Assessment, Diagnostic and Therapeutic Challenges

The diagnostic criteria for the 2019 NIH Expert Consensus must meet all five following conditions: (1) an increase in heart rate of more than 30 beats per minute (for adults) or more than 40 beats per minute (for individuals aged 12 to 19) within 10 min of standing or head-up tilt; (2) the absence of orthostatic hypotension (a drop of 20 mmHg or more); (3) frequent symptoms of orthostatic intolerance during standing, with rapid improvement upon returning to a supine position; (4) a duration of at least three months; (5) the exclusion of other conditions causing sinus tachycardia [4,102]. Reaching a threshold of HR > 120 beats per minute is no longer a criterion for the diagnosis. The diagnosis of POTS requires the exclusion of other conditions that could account for sinus tachycardia. However, it’s essential to recognize that certain conditions, including anxiety, pain, and deconditioning, can contribute to or exacerbate POTS. The potential overlap of symptoms and contributing factors across various medical conditions poses challenges in the diagnostic process. This complexity underscores the intricate nature of POTS and emphasizes the requirement for a comprehensive, patient-centered approach to diagnosis.

Due to its complex etiology, classifying POTS presents challenges. Despite the intricate etiological factors associated with POTS, its classification is invaluable for both clinical practice and research. It plays a crucial role in tailoring treatment strategies, facilitating effective communication, supporting clinical decision-making, and providing a framework for patient stratification. While acknowledging the dynamic nature of POTS and the overlap of contributing factors, a classification system ensures consistency in research studies and contributes to ongoing efforts to understand and manage this heterogeneous condition. We outlined a classification for the etiological factors in POTS as shown in Box 1, recognizing its critical importance for both clinical practice and research. It is important to recognize, however, that this classification is not definitive. As research in this area progresses, the classification should be subject to evolution and refinement. This adaptability is crucial, given the complexity of POTS and the diverse interactions and factors that may be present in individual patients. The goal is to continually enhance understanding and management of this condition, integrating new findings and perspectives as they emerge.

Box 1 **Classification of Etiological Factors in POTS.**

**Pathological Etiology:**
 1. Pathophysiological Factors:     ● Neurological/Pain-Related       ○ Autonomic Neuropathies       ○ Hyperadrenergic POTS       ○ Peripheral Neuropathy     ● Blood Volume-Related       ○ Thoracic Hypovolemia       ○ Diminished Venoconstriction     ● Muscular dysfunction        ○ Muscle Pump Dysfunction       ○ Reduced Cardiac Output 2. Immune/Inflammatory-Related     ● Autoimmune Responses     ● Inflammatory Mechanisms     ● Mast Cell Activation Disorders     ● Molecular Mimicry (e.g., post-HPV vaccination) 3. Connective Tissue Integrity:     ● Connective Tissue-Related       ○ EDS 4. Endocrine Factors:     ● Endocrine Dysfunction     ● Decreased Norepinephrine Clearance 5. Genetic/Hereditary:     ● Genetic/Hereditary Predisposition 6. Iatrogenic:     ● Medication-Induced 7. Pregnancy
**Non-Physiological Etiology:**
 1. Idiopathic POTS 2. Deconditioning 3. Psychological or Behavioral POTS     ● Anxiety/stress

Understanding the complexities of POTS is challenging, yet it offers significant opportunities for medical advancement. Medications such as beta-blockers, fludrocortisone, and midodrine can target the biological aspects by managing issues such as heart rate and thoracic hypovolemia. In this article, we cautiously bring attention to the sinoatrial node and myocardial function, particularly how the heart responds to stress despite exhibiting a normal cardiac phenotype at rest. Consistent with this notion, a major advancement in recent years is Ivabradine, which specifically targets the f-channels in cardiac pacemaker cells of the sinoatrial node by binding to the channel pore from the intracellular side [103]. These f-channels facilitate a slow, mixed Na^+^/K^+^ inward current that is crucial for pacemaker activity in the heart [104]. By modulating these channels, ivabradine may reduce heart rate without affecting blood pressure or myocardial contractility, therefore making it a promising therapeutic option for treating POTS. A systematic review by Gee et al. revealed that ivabradine effectively reduced heart rate and alleviated symptoms of POTS without affecting blood pressure [105]. Common side effects were dizziness, nausea, headache, and fatigue, which seldom necessitated discontinuation of the therapy [105]. In a recent randomized, double-blinded, placebo-controlled crossover trial, ivabradine significantly reduced heart rate and improved quality of life without significant side effects such as bradycardia or hypotension [106]. Alongside these clinical advances, the biopsychosocial model of health, integrating biological, psychological, and social dimensions [107], deserves careful consideration in this study of POTS due to its complex nature. Psychological therapies, including cognitive-behavioral therapy (CBT), support patients in managing the stress and anxiety related to POTS [108], while social interventions, such as patient education and support groups, are essential for addressing the social dimensions of the condition [109].

## 7. Conclusions and Perspective

POTS presents as a complex and enigmatic condition within the medical community, characterized by a perplexing array of symptoms revolving around orthostatic tachycardia. The etiology of POTS is multifaceted, involving disruptions in ANS activity and cardiac stroke volume regulation. This disorder can arise from a variety of factors, including difficulties in maintaining peripheral vascular tone, reduced blood volume, and abnormalities in heart rate regulation. The intricate interplay of these factors necessitates a comprehensive understanding of their individual and combined effects to fully comprehend the pathophysiology of POTS. To advance basic research, it is essential to recognize that POTS may co-occur with various underlying diseases. Exploring conditions with similar symptoms can provide valuable insights into potential contributing factors. However, the primary etiology of POTS, particularly in cases that occur in isolation without any discernible primary diseases, remains elusive. These idiopathic instances of POTS represent a significant gap in medical knowledge. Genomic approaches are proving to be powerful tools in identifying these underlying mechanisms and shedding light on the complexities of POTS etiology.

This review covers the etiology and the latest molecular insights into POTS. While progress has been made in understanding certain aspects of POTS, the overall picture of its origins remains complex and multifactorial. The continuing gaps in our understanding highlight the need for ongoing, dedicated research efforts. Such efforts are crucial for developing more effective and targeted therapeutic interventions, ultimately leading to improved patient outcomes and a deeper understanding of this perplexing syndrome. In line with these efforts, our proposed classification system for the etiological factors in POTS, as outlined in Box 1, represents an attempt to systematize the diverse aspects of this condition. By categorizing the various etiological factors and acknowledging the potential for co-occurring conditions, this classification aims to provide a clearer framework for both clinical and research perspectives. It is our hope that this contribution will facilitate a more structured approach to understanding POTS and inspire further research that builds upon this foundational work.

## Data Availability

Not applicable.
